# Distinct patterns of spread of prion infection in brains of mice expressing anchorless or anchored forms of prion protein

**DOI:** 10.1186/2051-5960-2-8

**Published:** 2014-01-21

**Authors:** Alejandra Rangel, Brent Race, Katie Phillips, James Striebel, Nancy Kurtz, Bruce Chesebro

**Affiliations:** 1 Laboratory of Persistent Viral Diseases, Rocky Mountain Laboratories, National Institute of Allergy and Infectious Diseases, Hamilton, Montana 59840, USA; 2 Rocky Mountain Veterinary Branch, Rocky Mountain Laboratories, National Institute of Allergy and Infectious Diseases, Hamilton, Montana 59840, USA

**Keywords:** Brain interstitial fluid, Cerebral amyloid angiopathy, Prion, Glycophosphatidylinositol anchor, Basement membrane, Transgenic mice

## Abstract

**Background:**

In humans and animals, prion protein (PrP) is usually expressed as a glycophosphatidylinositol (GPI)-anchored membrane protein, but anchorless PrP may be pathogenic in humans with certain familial prion diseases. Anchored PrP expressed on neurons mediates spread of prions along axons in the peripheral and central nervous systems. However, the mechanism of prion spread in individuals expressing anchorless PrP is poorly understood. Here we studied prion spread within brain of mice expressing anchorless or anchored PrP.

**Results:**

To create a localized initial point of infection, we microinjected scrapie in a 0.5 microliter volume in the striatum. In this experiment, PrPres and gliosis were first detected in both types of mice at 40 days post-inoculation near the needle track. In mice with anchored PrP, PrPres appeared to spread via neurons to distant connected brain areas by the clinical endpoint at 150 days post-inoculation. This PrPres was rarely associated with blood vessels. In contrast, in mice with anchorless PrP, PrPres spread did not follow neuronal circuitry, but instead followed a novel slower pattern utilizing the drainage system of the brain interstitial fluid (ISF) including perivascular areas adjacent to blood vessels, subependymal areas and spaces between axons in white matter tracts.

**Conclusions:**

In transgenic mice expressing anchorless PrP small amyloid-seeding PrPres aggregates appeared to be transported in the ISF, thus spreading development of cerebral amyloid angiopathy (CAA) throughout the brain. Spread of amyloid seeding by ISF may also occur in multiple human brain diseases involving CAA.

## Background

Prion protein (PrP) is a host-encoded membrane glycoprotein expressed in many tissues and cell types. The normal functions of PrP are not known, and PrPnull mice are for the most part clinically normal [1,2]. However, with sophisticated testing, PrPnull mice have detectable deficits in both neurophysiological and memory functions (for review see [3]). These functions appear to be related to the function of PrP in neurons because they can be reversed by expression of PrP in neurons using a neuron-specific transgene [4].

The most striking function of PrP to date is the absolute requirement for PrP expression for susceptibility of animals to prion infection and disease [1]. Disease-associated partially protease-resistant PrP (PrPres) is believed to be the infectious agent responsible for transmission between individuals [5-7], and the endogenous host PrP appears to interact with PrPres providing a substrate for new PrPres conversion [8]. In addition, host cellular PrP appears to be involved in transport of prion infectivity along peripheral nerves leading to the CNS [9-15] as well as along neuroanatomical pathways within the CNS such as the optic tract with multiple neurons and synapses leading from retina to the superior colliculus and optic cortex [14,16,17]. PrP expression on neurons or astrocytes is sufficient to meditate susceptibility and neuroinvasion by prion infectivity [17-20]. However, PrP expression on other cell types such as lymphocytes or hepatocytes does not lead to susceptibility to prion infection [21].

Although prion protein (PrP) is expressed mainly as a glycophosphatidylinositol (GPI)-anchored membrane protein, anchorless PrP is found in certain humans with familial prion disease and can be associated with pathology involving perivascular deposition of amyloid PrPres resulting in cerebral amyloid angiopathy (CAA) [22-26]. Expression of anchorless PrP in transgenic mice (tg44), results in susceptibility to intracerebral (i.c.) scrapie infection with replication of high scrapie infectivity titers and amyloid PrPres deposition in brain, heart, fat, colon, muscle and liver [27]. In brain there is extensive deposition of amyloid PrPres around blood vessels producing CAA pathology and clinical disease by 300–350 dpi [28]. This scrapie incubation period is much longer than the 150–160 day incubation period seen in C57BL/10SnJ (C57) mice expressing anchored PrP, which have vacuolar gray matter pathology and non-amyloid PrPres deposition, which is not associated with blood vessels [28]. The different incubation times and pathology seen after scrapie infection in mice expressing anchored versus anchorless PrP might be explained by differences in various aspects of the disease process including: first, different toxicity of amyloid and non-amyloid forms of prions; second, different susceptibility of mice to prion infection or damage; third, different ability to spread the infection to CNS locations resulting in clinical disease. In the present paper we describe experiments aimed at testing the latter two of these possibilities.

To investigate whether tg44 mice and C57 mice differed in susceptibility to scrapie infection, we compared these mouse strains in a limiting dilution titration of scrapie infectivity using i.c. injections. Surprisingly, the endpoint titer for scrapie infectivity in these two mouse strains was indistinguishable, suggesting that they were equally susceptible to the infection.

As another potential explanation for the difference in disease tempo in these mice, we next studied spread of scrapie PrPres within brain after intracerebral injection of each mouse strain. Previously we showed an association at the time of clinical disease between PrPres amyloid and the ISF drainage system using ISF tracers [29]; however, in this work we did not look at multiple preclinical time-points to determine the localization and pattern of spread of the PrPres amyloid over time. Because of the very poor spread of PrPres in peripheral nerves after intranerval injection of mice expressing anchorless PrP [30], we hypothesized that in brain of tg44 mice PrPres might not spread via neuroanatomical connections as seen in C57 mice. Two possibilities were considered: First, mechanical dissemination of PrPres aggregates capable of seeding new PrPres conversion might occur mostly at the time of intracerebral (i.c.) injection. Subsequent slow outgrowth of plaques from sites of original seed deposits would occur, but there might be negligible additional dissemination of new seeds. Alternatively, PrPres amyloid might spread continuously throughout the disease course via a slow pathway which did not require expression of anchored PrP on cell surfaces [31]. To limit the initial mechanical spread of the inoculum, we infected mice using intracerebral (i.c.) microinjection of scrapie in a small volume (0.5 μl), and at various times we studied the spread of PrPres throughout the brain by immunohistochemical evaluation. The results showed striking differences between mice expressing anchorless PrP versus anchored PrP, suggesting that unique mechanisms of spread of PrPres occurred in mice expressing anchorless PrP.

## Methods

### Ethics statement

All mice were housed at RML in an AAALAC-accredited facility in compliance with guidelines provided by the Guide for the care and use of Laboratory Animals (Institute for Laboratory Animal Research Council). Experimentation followed Rocky Mountain Laboratory Animal Care and Use Committee approved protocols 2010–08 and 2011–04.

### Mice

Transgenic mice were homozygous tg44+/+ mice expressing two alleles of a transgene encoding mouse prion protein lacking the GPI anchor on a genetic background of C57BL/10SnJ as described previously [28]. Mice were bred at RML. C57BL/10 mice were obtained from Harlan Sprague Dawley, Kent. WA, USA.

### Scrapie titration

To study sensitivity to mouse-adapted scrapie strain RML, mice from 4–6 weeks of age were inoculated intracerebrally with serial 10-fold dilutions from 10^-2^ to 10^-9^ in a volume of 50 μl. For intracerebral inoculation, mice were anaesthetized with isoflurane and injected manually in the left parietal lobe with a 27-gauge, 0.5-inch needle. Animals were observed daily for onset and progression of scrapie using previously described criteria [28]. The duration of disease varied from 30–60 days which was consistently longer than the duration noted in C57BL/10SnJ mice with anchored PrP. Typically in scrapie-infected Tg44+/+ mice, defective nesting was the first clinical sign. This was followed by hind limb weakness and a wide-based gait with low body posture. At later times severe hypoactivity and somnolence were noted. This condition was predictive of death within 1–2 days, and was designated as advanced clinical disease. Mice were euthanized when such signs were noted. Brains were removed and divided sagittally. One half was flash frozen in liquid nitrogen and kept at −80°C for future use in western blot analyses, and the other half was immersed in 10% neutral buffered formalin (3.7% formaldehyde) for histology.

### Intracerebral injection of FluoSpheres

In order to determine the extent of dispersion of a 50 μl injection into the brain of young mice, we injected 5 week old C57BL/10 mice intracerebrally with 50 μl of 1% (w/v) red fluorescent carboxylate-modified microspheres (0.02 μm; 580/505) (FluoSpheres, Molecular Probes, Eugene, OR). Mice were injected in the left parietal lobe. Other mice were microinjected stereotactically with 0.5 μl of FluoSpheres in the striatum. Five or 30 min after inoculation mice were anaesthetized with isoflurane, and then perfused intracardially with 0.1 M sodium phosphate buffer, pH 7.4 with heparin (30 units/ml) followed by 4% paraformaldehyde, adjusted to pH 7.3 by addition of NaOH. Brains were removed, post-fixed overnight in the same fixative, and cryoprotected in 30% sucrose in phosphate-buffered saline (PBS) without Mg^+2^ or Ca^+2^ for 48 h. Ten sagittal sections (20 μm thickness) were cut through the entire brain from the middle line using a cryostat (Leica, Richmond, IL, USA). Sections were placed on positively charged glass slides, and then air-dried overnight. Slides were hydrated in distilled H_2_O, and coverslips were applied to tissue sections using a mounting medium of Prolong Gold anti-fade reagent with DAPI reagent (Invitrogen).

### Stereotaxtic surgery

Intracerebral microinjections of 1% (w/v) RML brain homogenate stock were performed on adult male mice that weighed approximately 30 g. Age-matched transgenic tg44 and non-transgenic C57BL/10 mice were also injected with normal brain homogenate (NBH) in some experiments. Mice were anaesthetized with isoflurane and positioned on a stereotaxic frame (David-kopf Instruments, Tujunga, CA, USA). A 1-cm midline incision was made in the skin over the dorsal surface of the skull, and the skull was exposed to allow positioning of the drill over the Bregma point of reference. Co-ordinates used from Bregma were, +1 mm anteroposterior, +1.7 mm lateral and −3 mm ventral to skull surface. These co-ordinates were selected to target the center of the left striatum and avoid passing through any ventricle. Brain homogenates were injected with Nanofil syringes (World Precision Instruments, Sarasota, FL, USA) and steel bevel needles (32-gauge diameter) (World Precision Instruments) into the striatum at a rate of 0.25 μl/min with a total of 0.5 μl per mouse controlled with a pump (UltraMicroPump III with Micro4 pump controller, World Precision Instruments). The needle was kept in place for 2 min following injection to avoid the reflux of the brain homogenate solution. The skin incision was closed with suture. These conditions produced minimal brain damage and high reproducibility. Patency of the needles was verified prior to and after injections. Mice were euthanized by anesthesia overdose at different times after injection.

### Histology

Brains were removed and placed in 10% neutral buffered formalin for 3 to 5 days. Whole brains were divided coronally into 4 regions: (1) olfactory bulb to Bregma including the entire striatum, (2) middle thalamic area, (3) midbrain and (4) cerebellum. These tissues were then processed by dehydration and embedding in paraffin. The block including the striatum was cut into 5 μm coronal sections through the entire striatum region. Five adjacent sections were saved from each 75 μm linear region. One section from each group was stained by H&E to determine the needle track location. Additional sections from the other three areas (thalamus, midbrain and cerebellum) were obtained from mice at various time-points as needed. In early experiments each whole brain was embedded as a single block and 10 μm sections were cut, saving 4–5 adjacent sections out of each 100 μm distance. Sections were cut using a standard Leica microtome, placed on positively charged glass slides, and air-dried overnight at room temperature. The following day slides were heated in an oven at 60°C for 20 min and were then deparaffinized and rehydrated to aqueous conditions using standard procedures.

### Immunohistochemical detection of PrPres, GFAP and Iba1

Antigen retrieval and staining was performed using the Ventana automated Discovery XT stainer. PrP antigens were exposed by incubation in CC1 buffer (Ventana) containing Tris-Borate-EDTA, pH 8.0 for 100 minutes at 95°C. Staining for PrP was done using human anti-PrP monoclonal antibody D13 [32], which was obtained from tissue culture supernatants made in our laboratory from CHO cells expressing the D13 antibody construct, which were kindly provided by Dr. R. Anthony Williamson, The Scripps Research Institute, La Jolla, CA [29]. For immunohistochemistry, D13 culture fluid was used at a dilution of 1:100 for 2 hours at 37°C. The secondary antibody was biotinylated goat anti-human IgG at 1:500 dilution (Jackson ImmunoResearch, West Grove, PA.), and avidin-horseradish peroxidase was used with DAB as chromogen (DAB Map kit; Ventana Medical Systems, Tucson, AZ.).

Rabbit anti-Iba1 (Wako, Richmond, VA, USA) (dilution 1:2000), and rabbit anti-glial fibrillary acidic protein (GFAP) polyclonal antibody (1:3500) (Dako) were used. Antigen retrieval was done using CC1 buffer at 95°C for 44 min (Iba1) or 60 min (GFAP). Primary antibodies were diluted in PBS with 1% normal goat serum and 0.1% Triton X-100. Diluent without antibody was used as a negative control. Ventana streptavidin-alkaline phosphatase protocol was used to detect Iba1 and GFAP as described previously [33] with the exception that Fast Red chromogen was used. Slides were examined and photomicrographs were taken observed using an Olympus BX51 microscope and Microsuite FIVE software.

### Immunofluorescence staining

Slides were stained using the Dako Autostainer Plus machine (Dako, Carpentaria, CA, USA). Sections were permeabilized with PBS without Mg^+2^ or Ca^+2^ with 0.1% Triton X-100 for 15 min and blocked in 1% normal goat serum and 0.1% Triton X-100 in PBS for 15 min, followed by three 5 min rinses with PBS. For antigen retrieval of sections double-stained for PrPres plus CD31 or APP, tissue sections were soaked in citrate buffer, pH 6.0, and heated to 120˚C at 20 lb/in^2^ for 20 min using a BioCare decloaking chamber. For immunofluorescence staining, D13 supernatant was used at a 1:500 dilution, polyclonal rat anti-endothelial cell marker (CD31) antibody was used at a 1:50 dilution (Dianova, GmbH, Hamburg, Germany), and polyclonal rabbit anti-amyloid precursor protein (APP) was used at a 1:25 dilution (Invitrogen). Both primary antibodies were co-incubated for one hour at room temperature. This was followed by three 5 min rinses with PBS. Slides were then co-incubated with Alexa-Fluor 488 goat anti-rabbit Ig and Alexa-Fluor 568 goat anti-human Ig (Invitrogen, Carlsbad, CA) for 30 min at room temperature. Both secondary antibodies were used at a 1:200 dilution. Slides were next rinsed with double-distilled H_2_O, and coverslips were applied to tissue sections with ProLong Gold antifade reagent with 4’,6’-diamidino-2-phenylindole (DAPI; Invitrogen) to stain nuclei and protect fluorescence from fading. Diluent without primary antibody was used as a negative control. The staining was observed using an Olympus BX51 microscope using Microsuite FIVE software.

### Slide scanning

Aperio Digital Pathology Systems (Aperio Technologies, Vista, CA) was used to scan, capture, and store whole slide images from sagittal and coronal histology samples. Slides were scanned with the ScanScope® XT at 20× magnification for immunohistochemistry and ScanScope® FL for immunofluorescent samples. ImageScope™ software (Aperio ImageScope v10.1.3.2028) was used to view and export e-slide images in JPEG format.

## Results

### Comparison of limiting dilution scrapie titration in C57 versus tg44 mice

**Figure 1:**
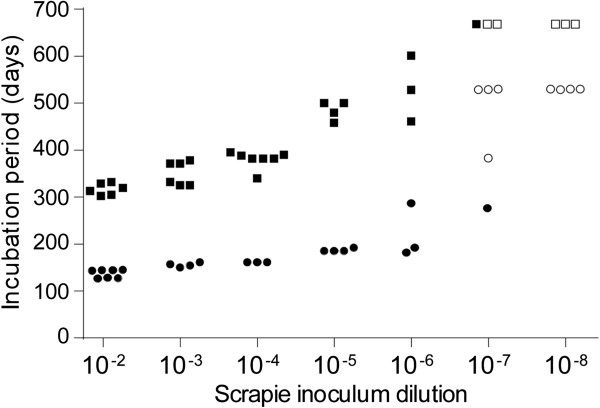
**Limiting dilution titration of RML scrapie in tg44 and C57 mice.** Serial ten-fold dilutions of RML scrapie were inoculated i.c. in a volume of 50 μl into either tg44 (squares) or C57 (circles) mice. Each point indicates the incubation period for one mouse; in cases of overlap the symbols have been separated to facilitate observation. Solid symbols indicate mice that had clinical signs and detectable PrPres in their brains. Open symbols indicate mice that did not develop disease or detectable levels of PrPres in brain. Using the data shown, the calculated titers for RML scrapie were: tg44 mice, 10^8.1^ ID50/gram of brain and C57 mice,10^7.9^ ID50/gram of brain.

In previous experiments i.c. injection of tg44 mice with 10^6^ ID_50_ of scrapie strains 22 L or RML resulted in clinical neurological disease at 300-350dpi, which was twice as long as the incubation period seen in non-transgenic C57 mice [28]. This might be explained by a difference between these mice in susceptibility to infection. To address this question in the present experiments, these mouse strains were compared by limiting dilution infection using a brain homogenate stock of RML scrapie made from C57 mice (Figure 1). Incubation period times were the time to advanced clinical disease, and the diagnosis of scrapie was confirmed by immunoblot detection of PrPres in brain. Nearly identical end-point dilution titers of 10^8.1^ and 10^7.9^ ID_50_/gram of brain in tg44 and C57 mice respectively were calculated from these data, indicating that these mice did not differ in susceptibility to scrapie infection. However, the tg44 mice had incubation periods which were 150–300 days longer than those seen in the C57 mice at each infectivity level tested (Figure 1). This difference appeared to correlate with the different types of pathogenesis induced by scrapie infection in these two types of mice [28].

**Figure 2:**
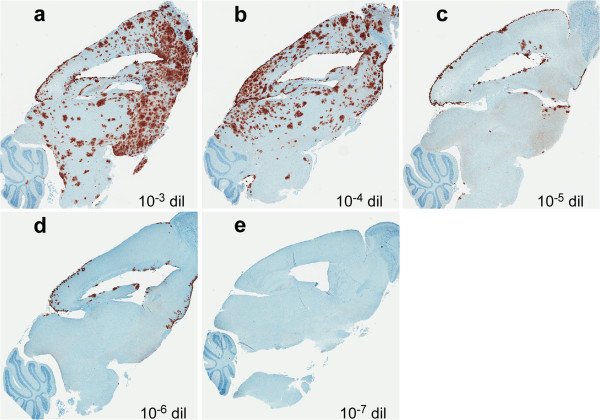
**Immunohistochemistry for PrPres in brains of tg44 mice following inoculation with different dilutions of RML scrapie.** Panels **a-e** show PrPres staining (brown) using anti-PrP antibody D13. Mice shown in panels **a-d** had clinical signs consistent with prion infection, whereas mouse in panel **e** did not have clinical signs and was euthanized at the end of the experiment (668 dpi). The dilution of inoculum and day of euthanasia for each panel are as follows: **a**: 10^-3^, 371 dpi; **b**: 10^-4^, 382 dpi; **c**: 10^-5^, 480 dpi; **d**: 10^-6^, 528 dpi; **e**: 10^-7^, 668 dpi.

In tg44 mice after inoculation of scrapie brain homogenate dilutions of 10^-3^ and 10^-4^, the time of clinical disease was 330–395 dpi (Figure 1), and immunohistochemical detection of PrPres in these mice revealed widespread plaques in most brain regions (Figures 2a and 2b). However, after inoculation of dilutions of 10^-5^ or 10^-6^,where the clinical endpoint was 458–601 dpi (Figure 1), fewer PrPres plaques were detected, and they were located mainly on meningeal and ependymal surfaces (Figures 2c and 2d). Since these areas are bathed in cerebrospinal fluid (CSF), these results suggested that a large part of the infection at these lower dilutions might have been initiated by infectivity entering the cerebrospinal fluid (CSF) at the time of injection, rather than by the injection into the brain parenchyma itself.

### Brain spread of i.c. injected FluoSpheres

**Figure 3:**
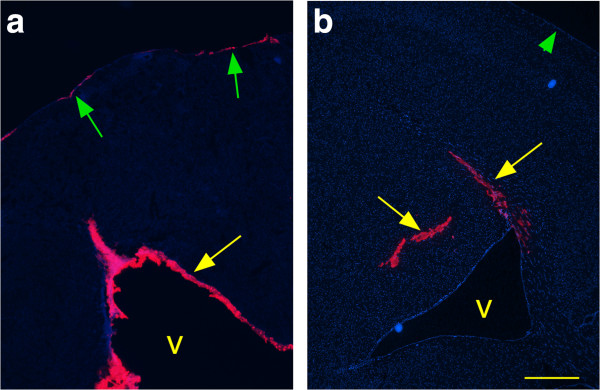
**Detection of 0.02 μm Fluospheres in brain at 30 minutes after i.c. injection. (a)** Injection of a 50 μl inoculum with a hand-held syringe. Pink Fluospheres were detected in ventricular (v) walls (yellow arrow) and on meninges (green arrows), but were not detected in brain parenchyma. **(b)** Microinjection of a 0.5 μl inoculum. Pink Fluospheres were detected in striatum and corpus callosum (yellow arrows), but not in ventricles (v) or on meninges (green arrowhead). Scale is same in each panel, and bar represents 500 μm.

The large injection volume (50 μl) used in the preceding experiments might result in spillover of the inoculum into the CSF during the injection. To test this possibility, FluoSpheres (size = 0.02 μm) were injected i.c. using either a 50 μl volume in a hand-held syringe or in a 0.5 μl volume using a microinjection method with a constant pressure pump over a 2 min period. Mice were euthanized 30 min post-injection for microscopic examination of brain tissue. After the 50 μl injection, FluoSpheres were mainly found associated with meningeal and ependymal surfaces (Figure 3a). In contrast, after microinjection of 0.5 μl, FluoSpheres were detected only within the brain parenchyma (Figure 3b). Similar results were seen in mice euthanized at 5 min post-injection. These data supported the interpretation that at the time of i.c. injection of a 50 μl volume the scrapie inoculum might easily contaminate the CSF leading to infection of meningeal and ependymal surfaces of the brain as was observed above in Figures 2c and 2d.

### Perivascular and extracellular spread of scrapie infection in CNS of tg44 mice

Based on the preceding experiments, to study the spread of scrapie infectivity within the CNS more precisely, we infected mice i.c. using stereotaxic microinjection into the striatum using a 0.5 μl volume of scrapie brain homogenate (400 ID_50_) over 2 minutes. With this method mice developed advanced clinical signs between 307–336 dpi. To study spread of scrapie within the brain, mice were euthanized at multiple time points, and coronal sections of the area of the striatum and other regions were studied by H&E staining for histopathology and by D13 anti-PrP staining using immunohistochemistry.

**Figure 4:**
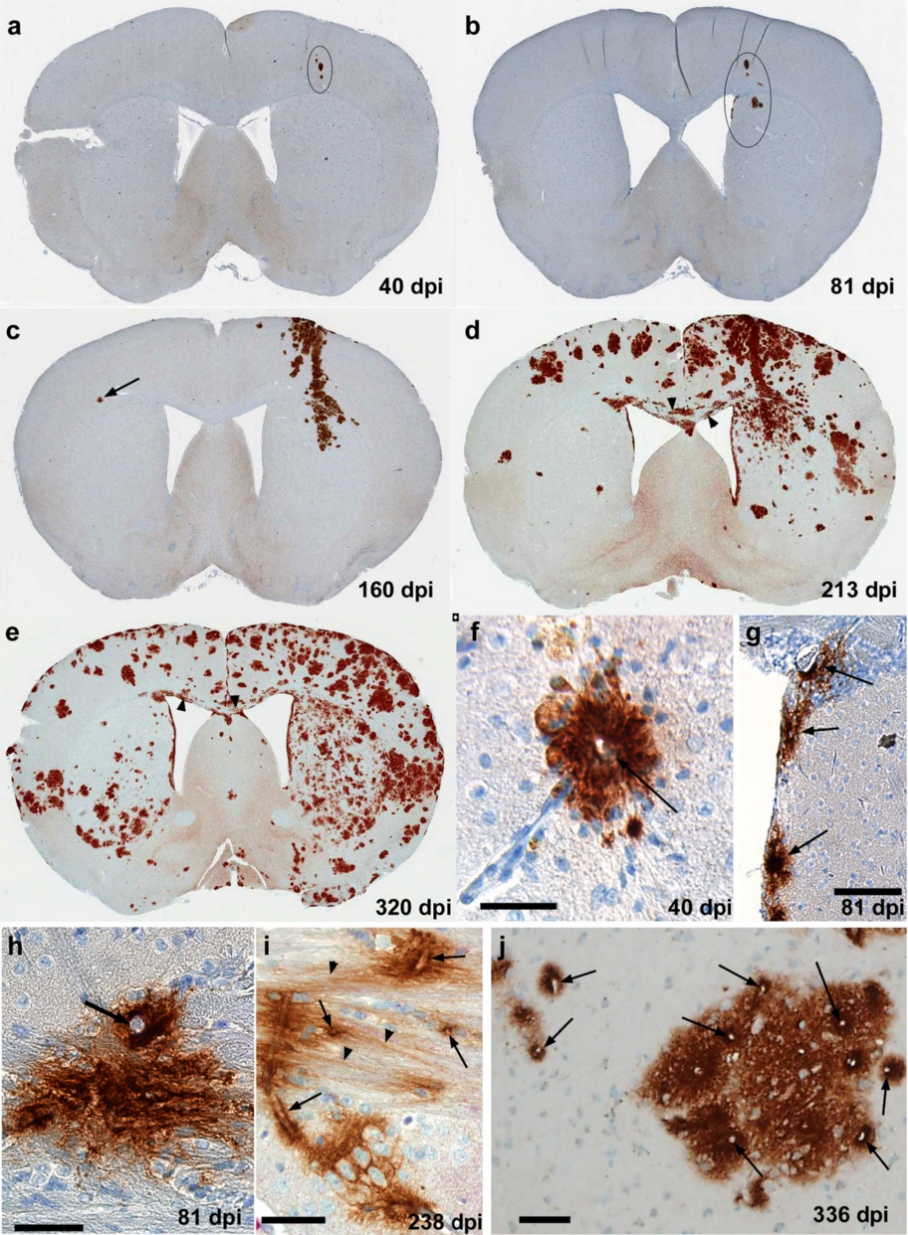
**Time course analysis of distribution of PrPres plaques in brain after microinjection of RML scrapie.** Panels **a-e** show detection of PrPres by Mab D13 in coronal sections of whole brain at the level of the needle track. **(a)** 40 dpi, PrPres plaques in needle track area of cortex are circled. **(b)** 81 dpi, several groups of plaques in cortex, striatum and adjacent subependymal area are circled. **(c)** 160 dpi, a larger area of PrPres staining can be seen in needle track region and neighboring areas from meningeal surface to striatum. Several groups of more isolated plaques can also be seen in adjacent areas. One plaque (arrow) is seen in cortex on contralateral side. **(d)** 213 dpi, numerous plaques are seen on both sides of brain, more in dorsal areas. Some streaked plaque staining can also be seen in the corpus callosum at higher magnification (arrowheads). **(e)** 320 dpi, plaques are found in most regions, but fewer plaques are seen in central ventral areas and in ventral cortex on contralateral side. At higher magnification streaming plaques in the corpus callosum can also be seen (arrowheads). **(f)** High power view of single perivascular plaque in cortex at 40 dpi from same mouse as panel **a**. Central blood vessel is shown by arrow. **(g)** Group of subependymal plaques (arrows) from same mouse as shown in panel **b**. **(h)** Single plaque with prominent vessel (arrow) at interface of corpus callosum and striatum at 81 dpi. **(i)** PrPres in corpus callosum associated with blood vessels (arrows) which are near to PrPres apparently streaming along interaxonal spaces (arrowheads). **(j)** Large plaque and adjacent small plaques in cortex with higher density of PrPres surrounding blood vessels (arrows). Scale bars are as follows: **f**, **h**, **i**, **j** = 50 μm; g = 100 μm.

After microinjection of scrapie, PrPres amyloid plaques were first seen at 40 dpi at the site of the needle track in the cerebral cortex (Figure 4a). At 81 dpi a few small plaques were seen near the needle track in the ipsilateral cortex, striatum, adjacent subependymal region and corpus callosum (Figure 4b). At 160 dpi, plaques were noted along the entire needle track in the cortex, corpus callosum and striatum (Figure 4c). Additional plaques were also noted in perivascular sites of gray matter and in extracellular spaces of corpus callosum near the needle track on the ipsilateral side. The first plaque on the contralateral side was detected in the cortex at 160 dpi (Figure 4c). At 213 dpi plaques were more widely distributed on both sides of the brain in many gray matter and white matter regions including the subependymal areas, but the ipsilateral side still had many more plaques than the contralateral side (Figure 4d). At 320 dpi the plaque distribution was even wider, and the two sides of the brain were nearly equal in distribution and density of plaques (Figure 4e).

Even at the earliest time points, 40 and 81 dpi, the plaques appeared to be associated with blood vessel walls and adjacent perivascular areas in both gray matter and white matter (Figures 4f- 4 h). Plaques in the subependymal regions appeared to associate with both the basal side of ependymal cells and with blood vessels (Figure 4g). At all time-points from 40–336 dpi, plaques were also seen in large white matter tracts such as the corpus callosum, and plaques also appeared to occupy extracellular periaxonal spaces (Figures 4h and 4i). In gray matter, even at late times such as 336 dpi, the smaller plaques on the contralateral side still maintained a consistent pattern of association with small blood vessels (Figure 4j), suggesting that perivascular spread was a common feature throughout the disease course. Thus, the timing and pattern of plaque distribution after microinjection of tg44 mice with RML scrapie supported our previous hypothesis that spread of infectivity occurred via dissemination with brain interstitial fluid (ISF) in perivascular regions of gray and white matter and in interaxonal spaces of white matter tracts [31].

### Vascular specificity at preclinical times after infection of tg mice

We have previously described the association of PrPres with capillaries, veins and arteries in tg44 mice at late times in disease after injection with 50 μl volumes of scrapie homogenate [31]. However, spread in these experiments might have been influenced by CSF contamination at the time of injection. In the current experiments we examined mice at earlier times after injection of 0.5 μl of scrapie to see whether PrPres was consistently associated with blood vessels at early and late times after microinjection. To confirm the association between perivascular blood vessels and PrPres in tg44 mice at preclinical times post-infection, brain tissues were double-stained for PrPres and for the blood vessel antigen, CD31, located in endothelial cell tight junctions of capillaries, veins and arteries.

**Figure 5:**
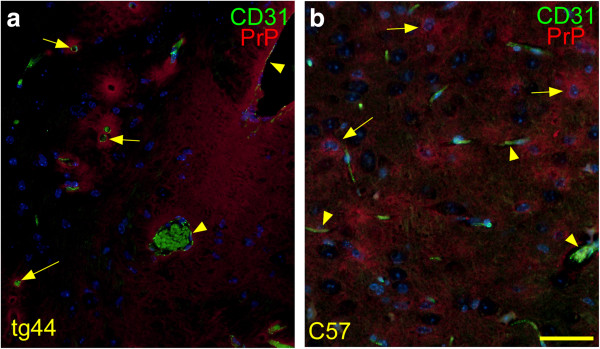
**Detection of PrPres and blood vessels in tg44 and C57 mice at 160 days after microinjection of RML scrapie.** Sections show thalamus at a level 2–3 mm caudal to the needle track. Sections were stained with D13 to detect PrPres (red), with anti-CD31 to detect blood vessel endothelial cells (green), and DAPI (blue) to detect nuclei. **(a)** In tg44 mouse dense PrPres plaques appear to associate with CD31-positive vessels of various sizes. Two large veins (arrowheads) and several capillaries (arrows) are indicated. Erythrocytes in vessels show green autofluorescence. **(b)** In C57 mouse PrPres is diffuse and appears to be concentrated around possible neurons (arrows) with large DAPI- stained nuclei, rather than around blood vessels (arrowheads). Scale is same in each panel, and bar represents 50 μm.

At 160 dpi tg44 mice had PrPres deposits associated with CD31-positive capillaries, arteries and veins (Figure 5a). As noted previously, the majority of PrPres was associated with capillaries, which were identified as blood vessels by their staining with anti-CD31 and as capillaries by their small diameters (3–7 μm). However, PrPres was also associated with larger vessels including both veins and arteries (Figure 5a) [31]. Similar results were seen at 81 dpi. This vascular PrPres distribution in tg44 mice differed markedly from the pattern seen in non-transgenic C57 mice expressing anchored PrP, where PrPres was not in a perivascular location, but instead tended to cluster around parenchymal cells with large nuclei, possibly neurons or astroglia (Figure 5b), as has been reported previously [34]. The data in tg44 mice supported our previous suggestion of a role for brain ISF in moving small diffusible PrPres oligomers to blood vessels where further conversion of new PrPres might be promoted by the presence of scaffolding molecules in basement membranes [31].

### Focal pathology associated with PrPres plaques in tg44 mice

**Figure 6:**
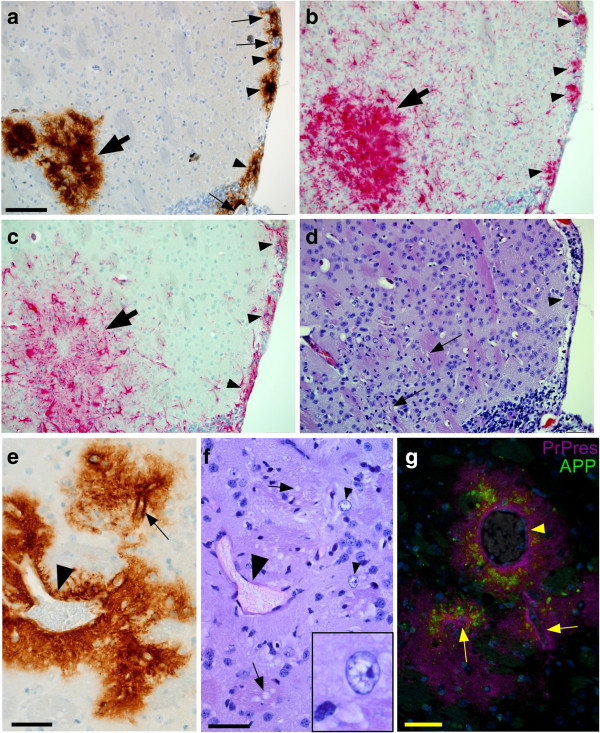
**Neuropathology in scrapie-infected tg44 mice at preclinical times. (a-d)** Panels show striatum and subependymal regions adjacent to needle track at 81 dpi. **(a)** PrPres visualized by D13 staining. Arrowheads show subependymal plaques often associated with blood vessels (arrows). On left side of field is a large plaque at the end of the needle track (bold arrow). **(b)** Staining with anti-Iba1 shows infiltration of microglia and/or macrophages often with an activated morphology consisting of larger cell bodies and thickened processes. Subependymal (arrowheads) and needle track (bold arrow) accumulations of cells correlate with PrPres plaques seen in **(a)**. **(c)** Staining with anti-GFAP shows accumulation of activated astroglia in areas of subependymal plaques (arrowheads) and needle track (bold arrow). **(d)** H&E staining of same field shown in **(a-c)**. In needle track area, arrows indicate small vacuoles in white matter of striatum. Near lower arrow hypercellularity and distorted architecture can be seen. Arrowhead indicates a subependymal plaque. Apparent vacuolation of subependymal region is likely an artifact. **(e)** Perivascular PrPres plaque in striatum of tg44 mouse at 160 dpi. A large vein (arrowhead) and prominent capillary (arrow) can be seen. **(f)** H&E staining of section in same area as **(e)** shows white matter vacuoles (arrows) and two nuclei with apparent vacuoles (arrowheads). Inset shows higher magnification of one nucleus with vacuoles. Large vein is indicated by larger arrowhead. **(g)** In striatum of same mouse shown in **(e)** and **(f)**, double-staining shows APP accumulation (green) associated with PrPres plaques (magenta) surrounding capillaries (arrows) and large vein (arrowhead). Scale is same in panels **a-d**, and bar shown in **(a)** represents 50 μm. Other bars are as follows: **e**, 65 μm; **f**, 25 μm; **g**, 50 μm.

At the clinical endpoint (300–330 dpi) in tg44 mice we previously found evidence of pathological changes around deposits of PrPres amyloid, including astrogliosis, microgliosis, distortion of neuroanatomy by amyloid, neuronal loss, white matter vacuolation, axonal dystrophy and deposition of amyloid precursor protein (APP) [28]. In these studies it was not possible to identify the earliest pathological changes because mice were examined only at the clinical timepoint. Therefore, in the present work we studied mice microinjected with scrapie at earlier times post-injection to attempt to identify the earliest pathological events. At 81 dpi, we found significant numbers of activated microglia and astroglia surrounding the newly formed PrPres plaques in striatum and subependymal regions (Figures 6a- 6c). In addition, morphological changes including vacuolation of white matter, foamy gray matter parenchyma, and occasional nuclear vacuoles, were also detected associated with PrPres deposits at 81–160 dpi (Figures 6d- 6f). These findings also correlated with detection of APP deposition associated with plaques (Figure 6g). However, no significant TUNEL staining or apoptotic bodies could be detected (not shown). Thus pathological abnormalities associated with plaques at early preclinical times in infected tg44 mice were similar to abnormalities seen previously at 330–350 dpi at the clinical end-point [28].

### Spread of scrapie infection in C57 mice expressing anchored PrP by non-vascular pathways

**Figure 7:**
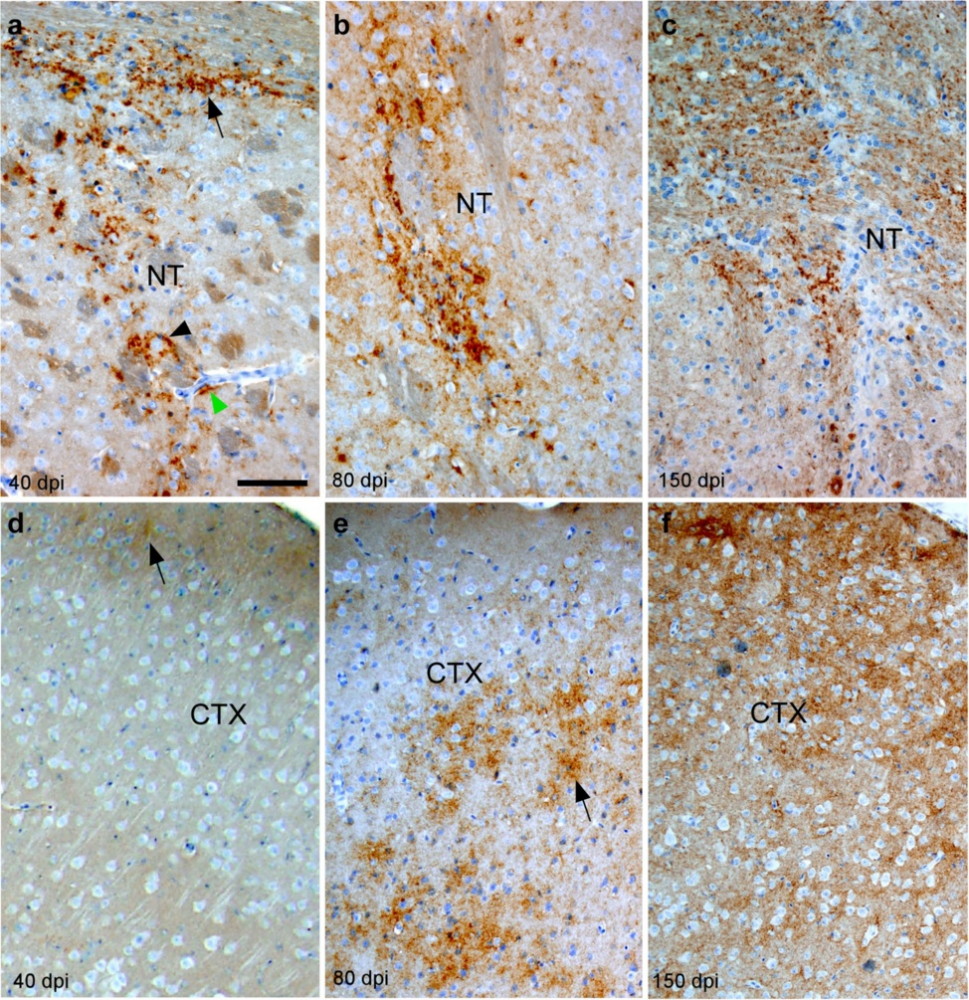
**Detection of PrPres by D13 staining in non-transgenic C57 mice at various times after microinjection of RML scrapie in striatum. (a)** At 40 dpi punctate PrPres staining can be seen in needle track in striatum associated with larger cells (black arrowhead) and in horizontally running axonal areas of corpus callosum at top of panel (arrow). Although blood vessels can be easily seen, there is minimal association of PrPres with blood vessels (green arrowhead). At 80 dpi **(b)** and 150 dpi **(c)** PrPres associated with needle track remains punctate and diffuse. **(d)** At 40 dpi in lateral cortex on ipsilateral side, there is faint PrPres staining (arrow) near outer surface of cortex. **(e)** At 80 dpi in contralateral cortex, prominent accumulation of diffuse and punctate PrPres (arrow) can be seen associated with parenchymal cells. **(f)** At the clinical disease end-point,150 dpi, diffuse and punctate PrPres occupies most of cortex. There is no obvious blood vessel association. NT: Needle track, CTX: Cortex. Scale is same in each panel, and bar shown in **(a)** represents 100 μm.

As a control for the above experiments with tg44 mice, C57 mice which express anchored PrP were also infected with scrapie i.c. using the microinjection technique. PrPres was detected in a non-amyloid diffuse form in these mice and was first seen at the needle track in striatum and corpus callosum at 40 dpi (Figure 7a), and only minimal staining was seen in cortex ispilateral to the needle track (Figure 7d). By 80 dpi, PrPres was seen in the striatum and ipsilateral cortex at sites near and more distant from the needle track (Figure 7b), as well as in contralateral cortex (Figure 7e). At 150–160 dpi at the clinical endpoint, PrPres was still visible near the needle track scar (Figure 7c), and in the contralateral cortex (Figure 7f), as well as in many other regions on both sides of the brain (not shown). This pattern of PrPres distribution did not appear to follow the blood vessels (Figure 5b) and was markedly different from the perivascular plaque pattern seen in tg44 mice (Figures 4 & 5a).

**Figure 8:**
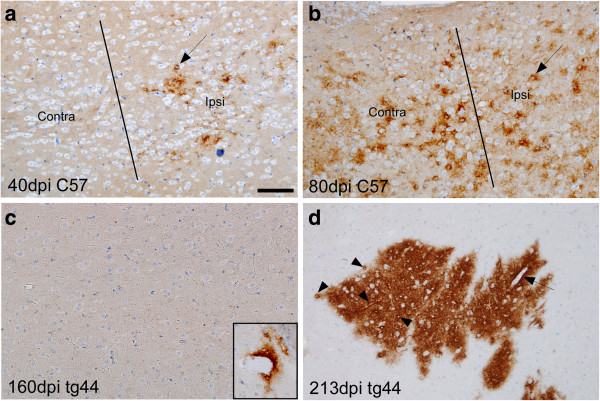
**Comparison of PrPres morphology at sites of secondary deposition in mid-thalamic region 3 mm caudal to the needle track injection site in C57 versus tg44 mice at various times after scrapie microinjection.** In C57 mice at 40 dpi **(a)** punctate PrPres is seen in thalamus on ipsilateral side but not on contralateral side. Line denotes middle of brain separating the two sides. Most PrPres is accumulated around cells with visible round nuclei (arrow). **(b)** At 80 dpi in a C57 mouse PrPres was detected on both sides of brain in thalamus, again mostly around cells with large nuclei (arrow). **(c)** At 160 dpi in tg44 mice no PrPres was detected at this level in thalamus. However, one perivascular plaque was seen in cortex in 1 of 3 mice (inset). **(d)** At 213 dpi in tg44 mice several PrPres plaques were seen in thalamus on both sides of brain. At higher power PrPres appeared to be concentrated around capillaries (arrowheads) and larger vessels (arrow). Scale is same in each panel, and bar shown in **(a)** represents 100 μm.

In addition, the apparent rate of PrPres spread from the inoculation site in striatum to thalamus at a level approximately 3 mm caudal to the needle track site was much faster in C57 mice than in tg44 mice. In Figure 8 spread of PrPres to ipsilateral thalamus was detected at 40 dpi and spread to contralateral thalamus was seen at 80 dpi (Figures 8a & 8b). In contrast, in tg44 mice the first detection of PrPres at this level was detectable in 1 of 3 mice at 160 dpi in cortex but not in thalamus (Figure 8c), and was first seen in thalamus at 213 dpi (Figure 8d). Thus after microinjection of scrapie into the striatum of C57 mice, the rapid spread of PrPres to thalamus and the cellular distribution within thalamus suggested that spread followed neuroanatomical pathways [35] rather than vasculature. In contrast, spread of PrPres to thalamus in tg44 mice was much slower and appeared to maintain its typical association with blood vessels as described above.

## Discussion

In the present experiments tg44 mice expressing anchorless PrP and C57 mice expressing anchored PrP were found to be equal in susceptibility when tested by limiting dilution i.c. infection using RML scrapie (Figure 1). Therefore, the differences previously noted between these mouse strains in scrapie incubation period, types of clinical signs and neuropathology [28] are likely due to factors other than susceptibility to infection. In tg44 mice the lack of carbohydrates and GPI anchor on the anchorless PrP molecules as well as the lack of attachment to the plasma membrane would all be expected to contribute to an increased susceptibility to amyloid formation during the generation of PrPres [8,36]. However, it is less clear whether the amyloid and non-amyloid forms of PrPres might differ in their neurotoxic potential. Furthermore, the cell surface expression of anchored PrPsen might also be an important factor in susceptibility of cells and tissues to toxic effects induced by PrPres and/or by disease-induced factors other than PrPres.

In addition to the actual mechanisms of brain damage induced by local prion infection and accumulation of PrPres, the ability of prion infectivity to spread within the brain would also be expected to influence the timing of clinical onset. Because of the slow course of prion infection, it might take significant time to spread the infection to brain areas vital for survival, such as brain stem and midbrain, where production of sufficient PrPres and other toxic products to induce significant dysfunction would also be required. Thus, in tg44 mice the long incubation period might also be due to slow spread from the site of injection to the rest of the CNS. In the present experiments, to measure spread of prions in tg44 and C57 mice, we used microinjection of scrapie infectivity in a 0.5 μl volume over 2 minutes to deposit the infectivity in the striatum with minimal leakage and dispersion via the CSF. With this method in tg44 mice PrPres was detected first at the injection site at 40 dpi and subsequently spread progressively to the rest of the brain over a total of 336 dpi (Figures 4a- 4e). The pattern of sequential IHC detection of PrPres indicated that the spread proceeded by following mostly along the perivascular regions of capillaries, arteries and veins in both gray and white matter, but also along interaxonal spaces in white matter tracts and in subependymal regions near ventricles (Figure 4f-j). In experiments using tracers of the brain ISF, all of these areas have been reported to be sites of ISF drainage [29,37-39]. Therefore our results suggested an important association between ISF drainage and PrPres amyloid deposition. Furthermore, PrPres association with ISF drainage areas was observed at 40-81 dpi, which was also the earliest times of PrPres detection (Figures 4f-h, and 5a). Thus the association of PrPres with the ISF drainage system was not merely a finding late in the disease process, but rather was an early event which might be important in the dissemination of PrPres through the brain. These results appeared to exclude the possibility that in microinjected mice dispersion of PrPres seeds occurred only at the time of initial injection, which was one possibility proposed in the introduction to this paper.

In our current experiments using microinjection of scrapie infectivity into C57 mice, spread of PrPres within the CNS was much faster than in tg44 mice. In C57 mice we detected spread of PrPres from striatum to ipsilateral thalamus by 40 dpi (Figure 8a) and crossover to the contralateral thalamus by 80 dpi (Figure 8b). This spread was much faster and the distance covered was much greater (3 mm from striatum to thalamus) than the limited PrPres spread detected in tg44 mice. In addition, in C57 mice there was minimal association of PrPres with blood vessels, but instead PrPres in C57 mice appeared to be concentrated around cells with medium to large nuclei which appeared to be neurons (Figure 5b). This localization and speed of spread was consistent with evidence from other experiments that in mice expressing anchored PrP scrapie could be transported rather rapidly along neurons following neuroanatomical pathways [9,35,40]. Thus, the differences in tempo and mechanisms of PrPres spread in tg44 versus C57 mice might contribute to the large difference in time of clinical onset in these two mouse strains.

Interestingly the pathological processes responsible for clinical scrapie disease are not identical in mouse strains differing in expression of anchored PrP [28], and these differences might also influence the timing of clinical disease onset. After scrapie infection, both tg44 mice and C57 mice have prominent astrogliosis and microgliosis. However, C57 mice have gray matter vacuolation together with deposition of non-amyloid PrPres in a diffuse pattern. In contrast, tg44 mice have perivascular deposition of PrPres in an amyloid form leading to cerebral amyloid angiopathy (CAA). Tg44 mice also show high amounts of APP co-localizing with PrPres suggesting axonal dystrophy, and they have prominent vacuolation of white matter, but not gray matter, suggesting a different type of brain damage than seen in typical prion disease. In the present experiments, from 81–160 dpi we found gliosis, white matter vacuolation and detection of APP associated with PrPres plaques in tg44 mice. This pathology was similar to the pathology previously described in clinical disease in these mice [28]. Thus, although local damage detected took less than 3 months to occur, 11–12 months was required for clinical onset, and this additional time might reflect the necessity for gradual spread of the infection and the pathology to wider areas of the CNS.

The present time-course experiments demonstrated slow spread of PrPres plaque deposition and related neuropathology associated with the ISF drainage system. A role for the ISF system in PrPres deposition in tg44 mice was first suggested by our earlier findings that PrPres deposits found at the clinical endpoint were associated with microinjected ISF tracers such as FITC-ovalbumin [29]. PrPres deposits were subsequently found to be associated with capillaries, arteries and veins in tg44 mice starting at the basement membrane [31]. Possibly basement membrane components, such as laminin or glucosaminoglycans, might bind small diffusible PrPres oligomers found in the extracellular fluid causing these oligomers to be concentrated and possibly assembled in ordered aggregates, thus facilitating their conversion to PrPres. Such a scaffolding mechanism might be more important for soluble anchorless PrP found in extracellular fluid compared to normal membrane-anchored PrP, which exists naturally in a partially concentrated array on the cellular plasma membrane. Finding of amyloid PrPres and associated pathology as early as 81 dpi in infected tg44 mice indicated that these processes proceed at a tempo nearly 4 times faster than the development of clinical signs, suggesting that the slow tempo of the clinical disease development is mainly due to the slow spread of the pathology to the rest of the brain.

Spread of PrPres via the ISF might also explain finding of PrPres mostly on meningeal and ependymal surfaces in tg44 mice injected i.c. with 50 μl volumes of highly diluted scrapie (Figure 2c and d). In this experiment the initial injection was probably distributed in large part via the CSF to meninges and ependyma. However, in most places the PrPres failed to penetrate from these sites deeper into the parenchyma, and this might be because the direction of ISF drainage flow is mainly along the perivascular regions going from the deeper brain areas towards the leptomeningeal areas where this flow ultimately exits the brain to enter the deep cervical lymph nodes [38,39].

In humans and animals expressing GPI-anchored PrP, spread of PrPres in both the CNS and peripheral nervous system appears to occur by following neuronal pathways [9,10,14,15,40]. In C57 mice we found a similar neuronal pattern of spread within the CNS after local microinjection of scrapie into the striatum. In our previous experiments using retinal injection of scrapie in a 2 μl volume, transgenic mice expressing anchored PrP only in neurons (tgNSE) showed spread of PrPres from retina to brain following the optic system, whereas mice expressing anchored PrP only in astrocytes (tgGFAP) showed very slow spread by a random cell-to-cell diffusing mechanism not involving nerves or blood vessels [17]. These data together with the present data in tg44 mice suggest that anchorless PrP was not sufficient for neuron-mediated spread of scrapie within the CNS and that presence of GPI-anchored PrP on neurons was probably required. The mechanism of neuron-mediated scrapie spread is not known, but its speed has been estimated to range from 0.5 to 3.3 mm/day [9,40,41], which is much slower than typical retrograde axonal transport, whose rate is from 85–430 mm/day [42]. In addition, conditions which hamper retrograde axonal transport such as overexpression of four-repeat tau, do not block neuronal transport of scrapie [43]. Therefore, spread of infectious prions along nerves probably involves a different type of mechanism [44,45]. Discovery of the details of this process should provide new approaches to control of clinical prion diseases in humans.

In humans four different mutations encoding PrP with a C-terminal truncation and lacking the GPI anchor (Y145X, Q160X, Y163X, and Y226X) have been discovered in patients with fatal neurodegenerative disease associated with perivascular amyloid PrPres deposition and CAA neuropathology similar to that seen in scrapie-infected tg44 mice [22-25]. Therefore, the lack of the GPI anchor might be important for development of this pathology. However, in an additional patient expressing anchorless PrP (Q227X), multicentric PrPres amyloid plaques were seen without evidence for CAA pathology [23]. Perhaps this mutant PrP is actually membrane bound by a mechanism not involving GPI, or alternatively, factors other than membrane binding of PrP might also be involved in promoting the generation of CAA.

The mechanism of spread of PrPres amyloid is not clear in any of these patients, but the similarity of the late stage pathology to our results in tg44 mice suggests that spread of amyloid conversion along the ISF drainage pathway might occur as a result of the secretion of anchorless PrP. Possibly pathogenic mechanisms similar to those seen in tg44 mice [28] might be involved in the tissue damage in these humans. CAA is also an important aspect of the pathology and pathogenesis of Alzheimer’s disease in humans, and the ISF drainage system might play a role in the spread of Aβ amyloid in this disease [46]. Thus scrapie-infected tg44 mice may provide a good animal model for study of mechanisms leading to CAA in both familial prion disease and Alzheimer’s disease. Further studies will be required to determine the details of the pathogenesis in both the human and animal systems.

## Conclusions

In scrapie-infected mice with anchored PrP, the disease-associated PrPres generated was mainly non-amyloid in structure and appeared to spread via neurons to distant connected brain areas where additional PrPres was generated by the clinical endpoint at 150 days post-inoculation. This PrPres was rarely associated with blood vessels. In contrast, in mice with anchorless PrP, PrPres was mostly in the amyloid form and spread did not follow neuronal circuitry, but instead followed a novel slower pattern utilizing the drainage system of the brain interstitial fluid (ISF) including perivascular areas adjacent to blood vessels, subependymal areas and spaces between axons in white matter tracts. The mechanism of spread of PrPres amyloid in transgenic mice expressing anchorless PrP appeared to be transport of small amyloid-seeding PrPres aggregates in the ISF, thus promoting the development of cerebral amyloid angiopathy throughout the brain. Spread of amyloid seeding by ISF may also occur in multiple human brain diseases involving cerebral amyloid angiopathy.

## Competing interests

The authors declare that they have no competing interests regarding data presented in this manuscript.

## Authors’ contributions

Experiments were done by AR, BR, KP, and NK. Data were analyzed by AR, JS, and BC. Paper was written by BR, JS and BC. All authors read and approved the final manuscript.
